# ARAapp: filling gaps in the ecological knowledge of spiders using an automated and dynamic approach to analyze systematically collected community data

**DOI:** 10.1093/database/baae004

**Published:** 2024-02-01

**Authors:** Alexander Bach, Florian Raub, Hubert Höfer, Richard Ottermanns, Martina Roß-Nickoll

**Affiliations:** Institute for Environmental Research, RWTH Aachen University, Worringerweg 1, Aachen 52074, Germany; Staatliches Museum für Naturkunde Karlsruhe, Erbprinzenstr. 13, Karlsruhe 76133, Germany; Staatliches Museum für Naturkunde Karlsruhe, Erbprinzenstr. 13, Karlsruhe 76133, Germany; Institute for Environmental Research, RWTH Aachen University, Worringerweg 1, Aachen 52074, Germany; Institute for Environmental Research, RWTH Aachen University, Worringerweg 1, Aachen 52074, Germany

## Abstract

The ARAMOB data repository compiles meticulously curated spider community datasets from systematical collections, ensuring a high standard of data quality. These datasets are enriched with crucial methodological data that enable the datasets to be aligned in time and space, facilitating data synthesis across studies, respectively, collections. To streamline the analysis of these datasets in a species-specific context, a suite of tailored ecological analysis tools named ARAapp has been developed. By harnessing the capabilities of ARAapp, users can systematically evaluate the spider species data housed within the ARAMOB repository, elucidating intricate relationships with a range of parameters such as vertical stratification, habitat occurrence, ecological niche parameters (moisture and shading) and phenological patterns.

**Database URL: ARAapp is available at**  www.aramob.de/en

## Introduction

In times of global and climate change, a better understanding of species-specific ecological demands is essential for understanding the ongoing species decline (1–5). Thus, in a first step, there is an urgent need to make existing distributed ecological information available in centralized structures to facilitate the ecological analysis of arthropod assemblages. For many species, however, little more than a few sentences on their ecology have been published, and these are often spread over past decades and are not easy to discover. The lack of species-specific ecological knowledge on a large scale is also addressed by the Hutchinsonian and Grinnellian shortfall (6–8). This is especially conspicuous among less prominent taxonomic groups, particularly arthropods, which are notably affected by the issue of ‘taxonomic bias’ (9). For arthropods, the use of centralized and publicly available databases offering ecological information is limited to a few groups (10–16). These databases primarily contain static trait values, like categorical values (e.g. food preference, size classes or flight ability) or values measured on the individual specimen (e.g. length of Femur I). However, with an appropriate data basis, ecological knowledge can also be generated in a dynamic data-driven approach (17). In this new paradigm, ecological knowledge, which traditionally has been obtained through laboratory experimentation, field observations and expert knowledge, is generated automatically and dynamically through the analysis of voluminous data using exploratory analysis techniques embedded in tailor-made analysis tools. The advantage of these automated applications is that the data volume, on which the analyses are based, steadily grows and, with appropriate assurance of data quality, a constant refinement of the results can be achieved. However, successful implementation of this methodology requires an adequate understanding of the targeted species group, associated specimen and data collection techniques, in addition to strict requirements regarding data quality, necessary to obtain meaningful results. Such an approach has already been shown for soil organisms (18) or for spatio-temporal biodiversity data (19, 20).

Embracing dynamic, data-driven ecological knowledge generation further holds significant promise in addressing knowledge gaps related to the newly introduced invasive species (21–24). For example, it enables early detection of habitats that may be particularly vulnerable, providing a valuable window for implementing timely and targeted intervention measures.

We utilized a recently operationalized European database (https://aramob.de/en) for systematically collected spider assemblage data to develop a tailor-made set of exploratory data analysis tools that are specifically designed to analyze habitat occurrence, phenology, companion species, vertical distribution and two ecological parameters (moisture and shading) of spiders. These tools enable the interactive evaluation of a database through user-friendly web interfaces, allowing for the efficient generation of dynamic ecological knowledge since this dearth of ecological knowledge serves as a significant impediment to comprehending spider diversity and implementing effective conservation measures in Europe (25).

## Methods

### The ARAMOB data repository

**Figure 1.**
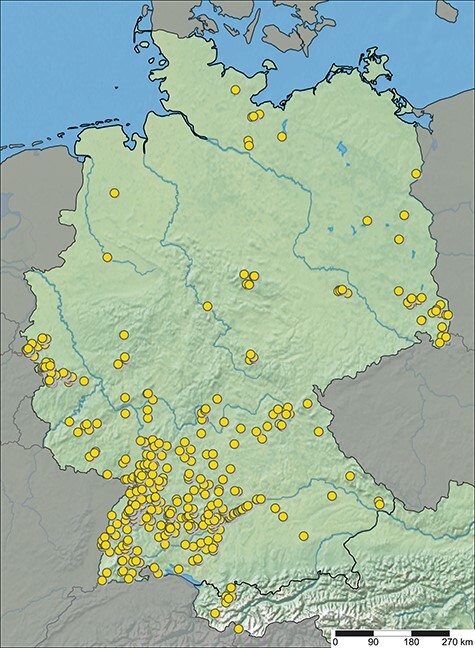
Sampling sites with spider assemblage data available in ARAapp, with data from 26 October 2023.

ARAMOB is a data repository for systematically collected spider assemblage data with standardized methods curated by the State Museum of Natural History Karlsruhe (Staatliches Museum für Naturkunde Karlsruhe) and the Arachnological Society for the German language area (Arachnologische Gesellschaft). The modular Diversity Workbench framework (26) is used to manage the data, which offers the advantage of facilitating standardization of data early in its life cycle through the specification of terminologies and ontologies, thus promoting the creation of **F**indable, **A**ccessible, **I**nteroperable and **R**e-Usable data (27–29). At present, the data repository encompasses assemblage data collected from over 1100 sampling sites, representing 107 distinct European Nature Information System (EUNIS) habitat types, primarily situated in Germany (Figure 1 and Table 1). This dataset encompasses 646 distinct species and ∼450 000 individual specimens (last visited: 26 October 2023).

**Table 1.** Distribution of sampling sites across first-level EUNIS 2012 habitat categories, with data from 26 October 2023

EUNIS habitat type	Sites with assemblage data
C: Inland surface waters	7
D: Mires, bogs and fens	18
E: Grasslands and lands dominated by forbs, mosses or lichens	408
F: Heathland, scrub and tundra	22
G: Woodland, forest and other wooded land	510
H: Inland unvegetated or sparsely vegetated habitats	41
I: Regularly or recently cultivated agricultural, horticultural and domestic habitats	22

### Data quality in ARAMOB

The assessment of biodiversity data quality necessitates a comprehensive evaluation of a set of quality metrics to determine its suitability for a specific purpose (30). This process comprises the consideration of three critical and interconnected components, namely, (i) the intended use, (ii) the relevant data type and (iii) the criteria employed to ascertain the data’s suitability in the intended context.

The intended use of the ARAMOB data repository is to furnish researchers with curated high-quality data packages to facilitate a cross-study analysis on spider assemblages to enhance species-specific ecological knowledge of spiders in Central Europe. To ensure the analyzability of community data across studies, it is essential to establish predefined data and metadata requirements. Given that pitfall trapping represents the most commonly employed method for studying spider assemblages, the following quality criteria are elucidated further, employing pitfall data as an illustrative example divided into three distinct categories: ‘Species’, ‘Methods’ and ‘Sites’.

The ‘Species’ identification must be done to species level, count data must be aggregated trap- or sampling-plot based to be spatial explicit (31). In the ‘Site’ section, a detailed exposition of the limitations pertaining to the sampling plots is provided. Foremost, it is imperative to ensure that species and individual counts are consistently attributed to specific habitats, ensuring a clear association between the data and their respective ecological and local contexts. Moreover, it is essential to exercise caution when dealing with highly aggregated data, such as information derived from country checklists, as they lack the requisite granularity for in-depth analysis and are therefore deemed unsuitable for further ecological analysis. It is also essential to methodologically separate species data collected using different methods (such as pitfall trapping and sweep-net sampling), to clearly attribute the resulting species count data to the corresponding method leading to a reduced bias in the analyses.

Although pitfall trapping is one of the oldest and most widely used systematic techniques for sampling ground-dwelling arthropods (based on their activity, therefore measuring ‘activity density’), just recently a first approach was published to standardize individual capture numbers across studies using the catch per unit effort (CPUE) (32). To implement this approach effectively, ‘methodological’ data must at least contain information about the number of pitfall traps deployed at each sampling site and the duration of sampling, to facilitate accurate calculation of CPUE values.

Accurately assessing the community structure and dynamics of a given ecosystem is essential for understanding its ecological processes and informing conservation efforts. In this regard, it is imperative to conduct habitat-based sampling to capture the full spectrum of heterogeneity within the system (33). Specifically, each sampling ‘Site’ (Plot) should be representative of a discrete habitat, devoid of any significant ecological gradients within it, to generate reliable and meaningful results (34, 35). To achieve this goal, the EUNIS habitat classification system (36) is utilized, which provides a comprehensive and standardized framework for categorizing habitats based on their unique ecological characteristics in Europe.

### Data pipeline

First, a data pipeline was developed in R (version 4.2.2) using RStudio to prepare the data using a knowledge discovery in databases approach (37, 38). After the export, the selected data undergo a series of preprocessing steps like standardizing fields with user-specific input (e.g. male/female, m/f) within the program routine to ensure consistency and homogeneity. The data cleaning step is an essential aspect, in which the data are systematically examined to ensure its quality and integrity. This step includes the assessment of data completeness and correctness by testing for the availability of the predefined criteria mentioned earlier. Additionally, validity and plausibility of taxonomic names is verified by comparing with the country list module of Araneae—Spiders of Europe (39). This query retrieves the current, valid species list for Germany and compares it to the taxonomies present in the data frame. Invalid species names are identified and recorded in an additional table for manual review by the data manager. As more data from other countries become available in the future, this review will be conducted depending on where the particular dataset was collected. This process ensures that the data are accurate and reliable and that any errors or inconsistencies are identified and addressed in a systematic manner. In the last step, count data are temporally and spatially normalized using the CPUE (32) to obtain comparable numbers of individuals across studies, and clean data tables are prepared for further applications.

### ARAapp description

The application is also written in R and uses shiny (40) and shinydashboard (41) packages to develop an easy-to-use graphical web interface. The application can be accessed via the ARAMOB site (www.aramob.de/en) with any modern browser. Running on a server, it eliminates the need for users to install R or download any additional software. After starting the application, the data pipeline is launched to export and prepare all available datasets, sampled with pitfall traps to the point where it is available for individual analysis. In the application, a selection list allows users to choose a spider species and various analysis tools. Depending on the selected tools and species, the prepared datasets are then filtered and undergo additional processing steps to match the necessary data aggregation level and requirements. This is particularly important when dealing with datasets that have varying temporal resolutions. In certain types of analyses, temporal resolution is not a crucial consideration and data that are only available in aggregated form over the entire collection time span can be utilized. In contrast, for other applications such as the study of phenological patterns, a high temporal resolution is essential. As previously mentioned, the data are initially exported and prepared by the data pipeline. Within the ARAapp, the data are further aggregated and filtered to ensure compatibility with the chosen analysis methods. Additionally, the users can set their own filters and restrictions on the data. At each analysis, all underlying processed data supporting the respective graph can be downloaded and utilized for further in-depth statistical assessments.

All graphs are built using the plotly package (42) and can be adjusted by zooming, scaling or manually hiding the data points. They can also be downloaded as a Portable Network Graphic and used for publications or other purposes. For each of the following tools, there is also a detailed manual, additionally supported by a general Frequently Asked Question secion available in the application.

## Results

### Analysis tools

Currently, there are five analysis tools available in the application. For each analysis, different quantity measures can be chosen, mainly the relative activity density (respectively, CPUE), the frequency value which is calculated as the percentage of sites in each class with a presence value for the chosen species and the percentage proportion of the selected species in its specific assemblage.

The ‘Companion species’ of the selected spider species are analyzed using the percentage share of spider species also sampled on the corresponding sites plotted on a horizontal bar chart. The user has the possibility to define the percentage threshold value manually with a slider widget. Furthermore, the user can restrict the trapping period to a specific time span to uncover temporal changes in communities.

**Figure 2.**
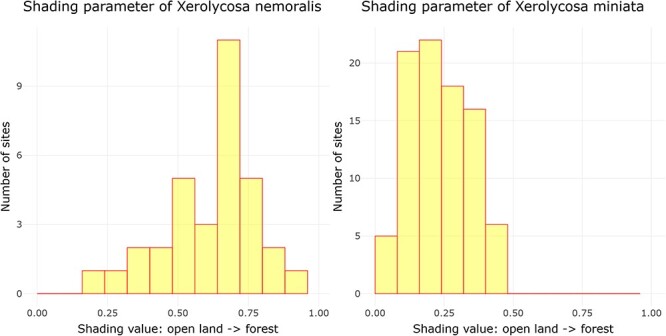
Distribution of the shading parameter values for *X. nemoralis* and *X. miniata*, and the graphs were downloaded on 26 October 2023.

Examining ‘Ecological parameters’, the next tool applies the methodology established by Entling *et al*. (43) to calculate shading and moisture parameter values for Central European spiders. This computation is solely based on the assemblage data, which is obtained from a carefully filtered dataset following the criteria outlined by Entling *et al*. (43). Initially, shading and moisture values are assigned to each sampling plot by conducting a correspondence analysis (CA) on the community data. The CA generates site scores, which are subsequently normalized between the range of 0 and 1, representing the first axis (shading) and the second axis (moisture) of the CA. The resultant parameter values, referred to as site scores, are then presented through a histogram visualization. In this histogram, the *x*-axis corresponds to the parameter values, while the *y*-axis represents the cumulative count of assemblages falling within each respective parameter width. This is illustrated in Figure 2 by the example of shading for two lycosid species with different shading requirements, *Xerolycosa nemoralis* (Westring, 1861), which prefers forest edges, and *Xerolycosa miniata* (C. L. Koch, 1834), which is inclined toward calcareous grasslands.

**Figure 3.**
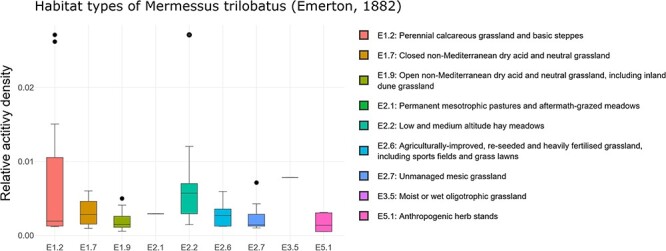
Habitat occurrence of *M. trilobatus* according to the EUNIS habitat classification at the third level within Class E: grasslands and lands dominated by forbs, mosses or lichens. The frequency data for the individual classes are as follows (sites with the presence of *M. trilobatus*/total sites in ARAMOB) E1.2: 12/86, E1.7: 6/14, E1.9: 15/26, E2.1: 1/6, E2.2: 13/28, E2.6: 14/24, E2.7: 8/35, E3.5: 1/12, E4.3: 7/69, E4.5: 3/46, and E5.1: 6/16. The graph was downloaded on 26 October 2023.

‘Habitat occurrence’ can be analyzed using box plots that illustrate the distribution of the selected species among EUNIS habitat types (currently available up to the fourth level, see Figure 3). By default, the top level of the EUNIS hierarchy is displayed first. To access the next lower levels, the corresponding level can be selected using the radio buttons, followed by the selection of the desired habitat category (e.g. E: grassland or G: forest) via the ‘Habitat Type’ dropdown list. Moreover, it is possible to apply data filtration based on the specific year of inquiry or the corresponding altitudinal range.

The ‘Vertical distribution’ of a species is shown on a horizontal bar chart where altitude meters are grouped in 100-m classes.

‘Phenology’ is analyzed using a scatterplot graph and is separated by gender. If the gender is not available, it is still shown as ‘unknown’ in the graph. In the program routine, the start and end dates of the sampling interval are used to calculate the date median, which is intended to minimize the error by considering a midpoint rather than either the start or end date alone. The graph legend allows the user to hide data for unknown genders if desired by clicking on the corresponding label. As a further filter, the altitudinal range of the plots used in the analysis can be restricted with the ‘Altitude’ slider.

Ensuring the reliability of generated results is of paramount importance, and as a preliminary gauge, all tools incorporate quality metrics. Each tool initiates the process by furnishing an overview of the database’s magnitude, indicating the extent to which the investigated species is represented across the maximum available sites. In some cases, this evaluation can be even more granular; for instance, the Habitat occurrence tools furnish an additional breakdown of the numerical distribution of investigated habitat types within the dataset, accompanied by specific instances of evidence for the studied species. Whenever feasible, it is recommended to evaluate these metrics to identify any potential disparities or imbalances within the data.

### Application example: Mermessus trilobatus

*Mermessus trilobatus* (Emerton, 1882) is a successful invasive spider species in Central Europe. Originating from North America, it was introduced to South Germany in the late 1970s (44). Since then, *M. trilobatus* has spread across most parts of Europe (45–48) and, unlike many other alien spiders that are synanthropically bound (49), it colonizes openland habitats (50, 51). Because the ecological requirements of alien species in newly established areas may differ significantly from those of their original habitats, understanding the factors that may promote the spread of this species is important to make early predictions about further dispersal. In Central Europe, it has taken 30–40 years since the first record until more extensive studies on the ecology of the species and possible drivers for the success of its colonization were carried out (50–54). Here, tools that automatically analyze large datasets can help to generate initial insights into the ecological requirements at an early stage, as well as the discernment of species or habitats that may be notably vulnerable. Currently, there are 130 sites with assemblage data available in the ARAMOB database with presence values of *M. trilobatus* in Germany from 2003 to 2020 (last visited: 26 October 2023).

An initial analysis with ARAapp indicates that *M. trilobatus* primarily thrives in EUNIS Habitat Type E: grasslands and lands dominated by forbs, mosses or lichens. Among the compiled assemblage data from 407 distinct sites, *M. trilobatus* was recorded in 22.4% of cases. It is noteworthy, however, that among the 508 available assemblage datasets from forests (EUNIS: G), *M. trilobatus* records are only discernible in six sampling sites. This limited occurrence suggests that the forests are unlikely to significantly contribute to the species’ spread. All other EUNIS habitat types are only sporadically represented in the ARAMOB database until now and thus will not be considered in the following analyses (Table 1).

Upon a closer examination of the grassland habitat types at EUNIS Level 3, it becomes evident that a diverse array of habitat subtypes, ranging from dry and mesic to both acidic and calcareous are colonized, spanning from lowland to alpine regions (Figure 3). Furthermore, it is also evident that not only disturbed or anthropogenically transformed habitats, but also semi-natural habitats like calcareous grasslands are invaded. This finding fortifies the hypothesis that, alongside high dispersal behavior (55), low habitat specificity (51) plays a crucial role in the invasion success.

While invasive linyphiids have demonstrated the ability to effectively compete with native counterparts (56, 57), such competitive behavior has not been documented for *M. trilobatus* yet (52). Utilizing the Companion species analysis tool, it is possible to discern species that may pose a considerable risk of competition with *M. trilobatus* in future. Four species belonging to the Linyphiidae family were identified, based on their equal body size and similar ecology, co-captured with *M. trilobatus* at a frequency exceeding 0.5. In decreasing order, these are *Erigone dentipalpis* (Wider, 1834), *Tenuiphantes tenuis* (Blackwall, 1852), *Erigone atra* (Blackwall, 1833) and *Agyneta rurestris* (C. L. Koch 1836). These findings align with the literature (50), underscoring the potential significance of these identified Linyphiidae species as potential competitors with *M. trilobatus* in future ecological scenarios.

In conclusion, the application of the developed ecological analysis tools was demonstrated to be an effective complementary method for conventional studies. While in case studies only limited sampling sites could be analyzed (51), those tools offer valuable supplementation. Regarding *M. trilobatus*, the analysis of the ARAMOB data suggests that the ability to colonize multiple habitat types could be a contributing factor to its rapid spread in Central Europe.

## Discussion

One of the main benefits of ARAapp is its ability to allow researchers to visualize and analyze the large datasets quickly and easily. This can save time and resources compared to more traditional data analysis methods while considering that the tools do not generate statistically valid results. These must be performed in a downstream process, such as downloading the processed data in the tools or the raw data via the ARAMOB portal. With the expansion of the database, a more comprehensive understanding of the ecological requirements of spider species becomes attainable. As exemplified by Entling *et al*. (43), the ecological requirements of the spiders regarding moisture and shading in the habitats are limited to only approximately half of the spider species found in Germany. However, through automated evaluation facilitated by a growing dataset, previously unstudied species can be subjected to enhanced ecological assessments. As the foundational data increases, these species can be more effectively characterized and their ecological attributes better comprehended, thereby contributing to a more holistic understanding of spider ecology. Overall, the results show that the tools programmed here are well suited to automatically visualize (aut-)ecological information from curated datasets. This kind of applications is especially useful for non-species specialists as the information is provided in an easily accessible web portal. For example, with the appropriate underlying data, initial analyses of newly introduced species can be performed as long as scientific studies with more detailed analyses on the specific species are lacking. This supports authorities or nature conservation organizations (58, 59). The utilization of a data management system, such as Diversity Workbench, during the data life cycle within the ARAMOB data repository further ensures a high level of standardization. This standardization enables the efficient adaptation of the application to other organism groups. Particularly, organism groups that are sampled using the same method, such as ground beetles (Coleoptera: Carabidae), rove beetles (Coleoptera: Staphylinidae) or woodlice (Isopoda: Oniscidea), can benefit from the quick transferability of the application. Although other methods like arboreal eclectors can be integrated without further ado, the extent to which the individual count numbers generated with these methods can be standardized across studies must still be examined.

Despite its many benefits, ARAapp does have limitations that researchers should be aware of. The main limitation is that they are only as good as the data they are based on. Even though the ARAMOB database is a reliable source of research-quality data on spider assemblages, it is still possible for bias to be present in the data. The most obvious bias here is of course the sampling method ‘pitfall trap’, which primarily captures epigeic spider species (60) due to their activity. This means that species that build stationary webs or ambush or those that live and hunt within the herb layer are not accurately represented (i.e. with a bias to males) or even completely absent from the data. Therefore, quality indicators such as the number of available datasets should always be checked to evaluate the significance of the respective tools. The same applies to the over- or under-representativeness of different habitat types. While underrepresented habitats are, due to standardization, less problematic in species count-based analyses (aside from the small sample size), they often dominate frequency-based analyses. It is therefore essential to carefully evaluate the data quality indicators for sample size given in the respective analysis when interpreting the results.

Finally, this article aims to serve as an impetus for European researchers to actively contribute their systematically collected spider data to the ARAMOB data repository, thereby facilitating its availability for comprehensive analyses.

## Data Availability

The basic code of the application is available on GitHub (https://github.com/alexander-bach/ARAapp/), excluding the Structured Query Language query from the database and initial preprocessing steps necessitated by the database structure for security reasons. However, the application can be run with own data. Three tables are required, which are also provided as a template on GitHub. The first table, ARAMOB_data, encompasses all species, method and sampling plot relevant data compiled in the previously prepared data pipeline and can be filled with own data. The subsequent tables, EUNIS_list and TRAIT_list, are publicly available lists obtained from the Diversity Workbench framework. EUNIS_list facilitates the breakdown of EUNIS codes into habitat descriptions, while TRAIT_list comprises a comprehensive inventory of available spider traits.
